# Supplementary material for: Risk Factors for Perioperative Urinary Tract Infection After Living Donor Kidney Transplantation Characterized by High Prevalence of Desensitization Therapy: A Single-Center Analysis
Source: J Clin Med. 2025 Aug 28;14(17):6102. doi: 10.3390/jcm14176102 (PMC12429468; doi:10.3390/jcm14176102)
Supplement: Supplementary file 1 [file jcm-14-06102-s001.zip › jcm-3825316-supplementary.pdf]

Table S1. Profiles of asymptomatic pathogens postoperatively isolated.

Urine samples	Number of isolates
Enterococcus feacalis	8
Escherichia coli	2
Escherichia coli (QREC)	1
Escherichia coli (ESBL)	2
Klebsiella aerogenes	3
Klebsiella pneumoniae	2
Klebsiella oxytoca	1
CNS	2
MRCNS[a]	2
Others: Gram-positive bacteria	3
Gram-negative bacteria	5

[a]MRCNS: Methysirin resistant coaglase-negative staphyococci

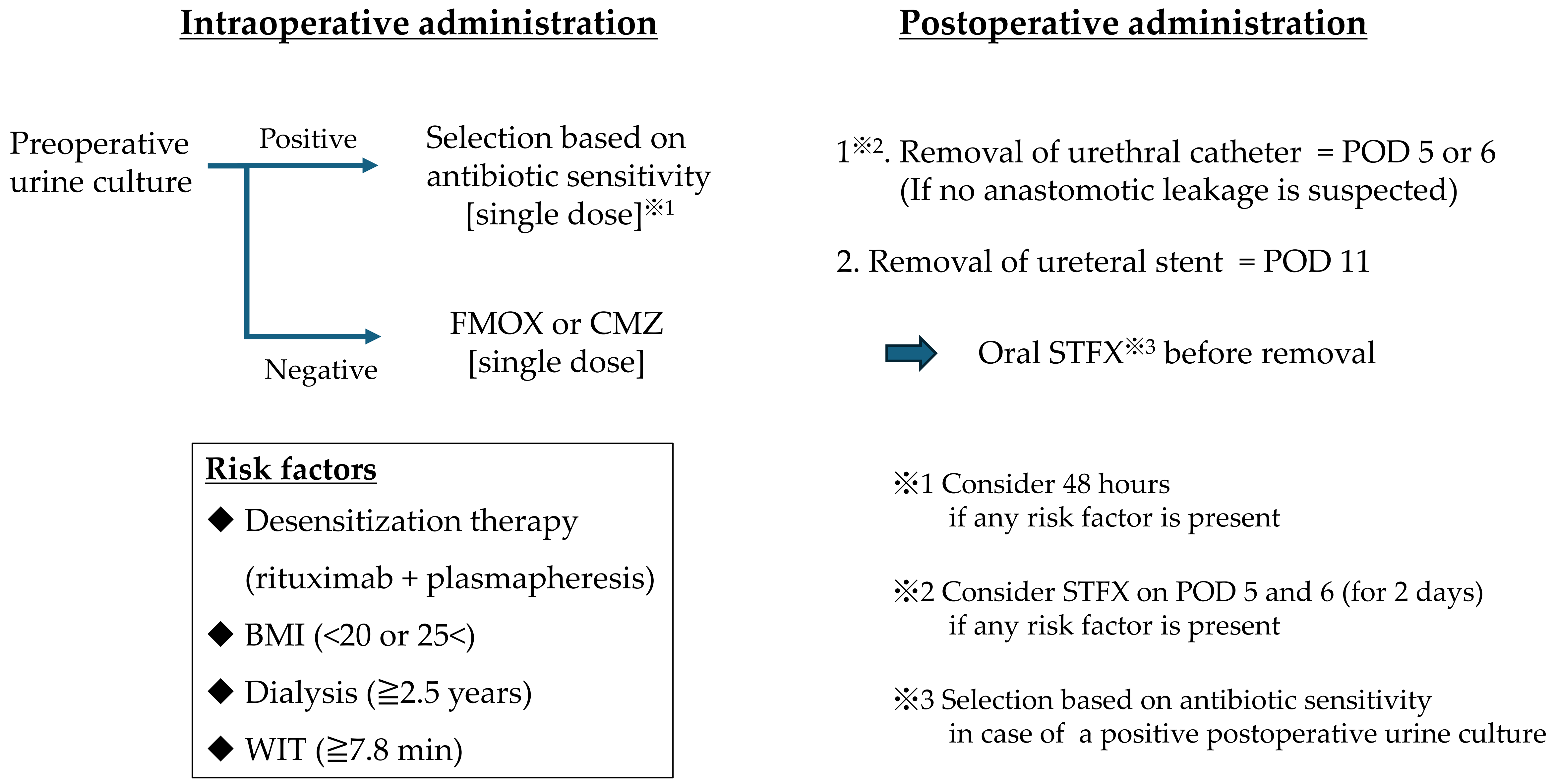

Figure S1. Draft Protocol for Perioperative Prophylactic Antibiotics in LDKT
